# Relay oral therapy in febrile urinary tract infections caused by extended spectrum beta-lactamase–producing Enterobacteriaceae in children: A French multicenter study

**DOI:** 10.1371/journal.pone.0257217

**Published:** 2021-09-16

**Authors:** Gabriel Lignieres, André Birgy, Camille Jung, Stéphane Bonacorsi, Corinne Levy, François Angoulvant, Emmanuel Grimprel, Marie Aliette Dommergues, Yves Gillet, Irina Craiu, Alexis Rybak, Loic De Pontual, François Dubos, Emmanuel Cixous, Vincent Gajdos, Didier Pinquier, Isabelle Andriantahina, Valérie Soussan-Banini, Emilie Georget, Elise Launay, Olivier Vignaud, Robert Cohen, Fouad Madhi

**Affiliations:** 1 Service de Pédiatrie Générale, Centre Hospitalier Intercommunal de Créteil, Créteil, France; 2 Service de Microbiologie, CHU Robert Debré, Centre National de Référence (CNR) associé *Escherichia coli*, Paris, France; 3 Centre de Recherche Clinique (CRC), Centre Hospitalier Intercommunal de Créteil, Créteil, France; 4 GPIP (Groupe de Pathologie Infectieuse Pédiatrique) de la SFP (Société Française de Pédiatrie), Paris, France; 5 ACTIV, Association Clinique et Thérapeutique Infantile du Val-de-Marne, Créteil, France; 6 Université Paris Est, IMRB-GRC GEMINI, Créteil, France; 7 Service des Urgences Pédiatriques, CHU Necker, Paris, France; 8 Service de Pédiatrie Générale, CHU Trousseau, Paris, France; 9 Service de Pédiatrie Générale, Centre Hospitalier de Versailles, Versailles, France; 10 Service des Urgences Pédiatriques, CHU Lyon, Lyon, France; 11 Service des Urgences Pédiatriques, CHU Bicêtre, Le Kremlin-Bicêtre, France; 12 Service des Urgences Pédiatriques, CHU Robert Debré, Paris, France; 13 Service des Urgences Pédiatriques, CHU Jean Verdier, Bondy, France; 14 Service des Urgences Pédiatriques, CHU Lille, Lille, France; 15 Service des Urgences Pédiatriques, Centre Hospitalier de Roubaix, Roubaix, France; 16 Service de Pédiatrie Générale, CHU Béclère, Clamart, France; 17 Service de Pédiatrie Générale, CHU Rouen, Rouen, France; 18 Service de Pédiatrie Générale, Centre Hospitalier de Saint-Camille, Bry sur Marne, France; 19 Service des Urgences Pédiatriques, CHU Ambroise Paré, Boulogne Billancourt, France; 20 Service de Pédiatrie Générale, Centre Hospitalier de Villeneuve-Saint-Georges, Villeneuve-Saint-Georges, France; 21 Service de Pédiatrie Générale, CHU Nantes, Nantes, France; 22 Service de Pédiatrie Générale, Centre Hospitalier de Meaux, Meaux, France; 23 Unité Court Séjour, Petits nourrissons, Service de Néonatalogie, Centre Hospitalier Intercommunal de Créteil, Créteil, France; Nitte University, INDIA

## Abstract

**Objectives:**

We need studies assessing therapeutic options for oral relay in febrile urinary tract infection (FUTI) due to ESBL–producing Enterobacteriaceae (ESBL-E) in children. Amoxicillin-clavulanate/cefixime (AC-cefixime) combination seems to be a suitable option. We sought to describe the risk of recurrence at 1 month after the end of treatment for FUTI due to ESBL-E according to the oral relay therapy used.

**Materials and methods:**

We retrospectively identified children <18 years who were included in a previous prospective observational multicentric study on managing FUTI due to ESBL-E between 2014 and 2017 in France. We collected whether children who received cotrimoxazole, ciprofloxacin or the AC-cefixime combination as the oral relay therapy reported a recurrence within the first month after the end of treatment. Then, we analyzed the susceptibility drug-testing of the strains involved.

**Results:**

We included 199 children who received an oral relay therapy with cotrimoxazole (n = 72, 36.2%), ciprofloxacin (n = 38, 19.1%) or the AC-cefixime combination (n = 89, 44.7%). Nine (4.5%) patients had a recurrence within the first month after the end of treatment, with no difference between the 3 groups of oral relay (p = 0.8): 4 (5.6%) cotrimoxazole, 2 (5.3%) ciprofloxacin and 3 (3.4%) AC-cefixime combination. Phenotype characterization of 249 strains responsible for FUTI due to ESBL-E showed that 97.6% were susceptible to the AC-cefixime combination.

**Conclusions:**

The AC-cefixime combination represents an interesting therapeutic option for oral relay treatment of FUTI due to ESBL-E as the recurrence rate at 1 month after the end of treatment was the same when compared to cotrimoxazole and ciprofloxacin.

## Introduction

Febrile urinary tract infections (FUTIs) are the most common proven bacterial infections among infants and young children presenting fever without a source [1–3]. FUTIs are most frequently due to infection with *Enterobacteriaceae*, mainly *Escherichia coli* [4, 5]. The emergence of ESBL-producing Enterobacteriaceae (ESBL-E) as a cause of FUTI presents a serious threat to public health given that therapeutic options are limited [6–8].

The main international guidelines recommend prescription of an oral antibiotic for FUTI [9–11]. Parenteral treatment is required only for children who are severely ill or unable to retain oral intake. These guidelines suggest that FUTI treatment last 7 to 14 days. This broad range is due to the lack of sufficient data identifying the optimal treatment duration [12]. However, because ESBL strains also frequently harbor resistance genes to cotrimoxazole and quinolones, no oral compound is suitable to treat FUTI due to ESBL-E. Thus, a sequence treatment is recommended in these children. Because an intravenous antibiotic treatment active for ESBL-PE is prescribed in many centres, oral antibiotic relay is necessary afterward to reduce the total duration of hospitalization after checking the strain’s antibiotic susceptibility. All the more so as most children are apyretic after a mean duration of antibiotic treatment of 2 to 4 days. This observation underscores the major value of using early oral treatment for this type of infection.

Indeed, children with a diagnosis of FUTI receive empirical treatment with broad-spectrum cephalosporins such as ceftriaxone or cefotaxime intravenously or cefixime orally. Some teams use aminoglycosides as first-line treatment [13]. After receiving the drug susceptibility testing (DST) results on urine culture, the treatment is switched to antimicrobials to which the causative organism is susceptible [2].

In a previously published prospective cohort, cotrimoxazole and ciprofloxacin were the most frequently used antibiotics for oral relay therapy in FUTI due to ESBL-E in children [14]. The non-orthodox amoxicillin-clavulanate and cefixime (AC-cefixime) combination was given to 86 (31.3%) children [14]. The short-term evolution was similar whatever the efficacy of the empirical treatment [14]. We sought to describe the risk of recurrence at 1 month after the end of treatment of the infectious episode according to the oral relay used (cotrimoxazole, ciprofloxacin or the non-orthodox AC-cefixime combination) in that same cohort. In a second step, we studied the susceptibility of ESBL-E strains on DST results to the AC-cefixime combination and to a large number of other antibiotics.

## Materials and methods

### Study design and population

We retrospectively identified children from the hospital-based active surveillance of FUTI due to ESBL-E created by the GPIP/ACTIV network. These children were included in the first part of a prospective observational study between March 2014 and March 2017 on behalf of the FUTI National Observatory due to ESBL-E in children, which was previously published [14]. Inclusion and non-inclusion criteria, demographic features of patients and clinical outcomes were previously described [14].

For this second analysis, all children with FUTI (as defined in the French recommendations) [15] due to ESBL-E were enrolled if the oral relay treatment (after empirical and definitive treatment) was cotrimoxazole, a fluoroquinolone or the non-orthodox AC-cefixime combination. Patients were excluded from the final analysis if their parent’s email, address or telephone number could not be found or with no response to the interviewer’s emails and calls.

We sent parents an email or a letter describing and explaining the reasons and expected results of our study and informing them of an upcoming phone call. Patients were given the opportunity to decline to participate in the study prior to the phone call, by return email to dpo@chicreteil.fr or letter. All legal guardians of included children provided oral informed consent.

Parents were then invited to a telephone interview of up to 10 min, during which they were asked “Did a recurrence occurred within the first month following the end of treatment of the initial FUTI due to ESBL-E?” If the answer was no, the interviewer ended the call. If the answer was yes, the investigator sought additional information: the precise diagnosis of the recurrence (cystitis or FUTI), which bacteria was isolated (with or without ESBL resistance mechanism), and whether the recurrence required antibiotic treatment, and if so, which one. In the event the parents did not remember the information mentioned above, the interviewer asked them to search for it in their child’s “health record”. If they did not have the health record at the time of the interview, a new telephone appointment was scheduled. Following the telephone call, the patient’s participation in the study is documented in his or her medical record by the investigator.

### Microbiology

ESBL-E strains identified during the first published study were sent to the *E*. *coli* National Reference Center (NRC) laboratory at Robert Debré Hospital (Paris, France) for further phenotype characterization. In brief, one colony of each morphologic type growing on the medium was identified by using the API20E system (bio Mérieux, Marcy l’Etoile, France) or with the Bruker Biotyper Matrix-Assisted Laser Desorption Ionization‒Time of Flight Mass Spectrometer. Antibiotic susceptibility was determined by using the disc diffusion method on Mueller-Hinton agar and interpreted as specified by the European Committee on Antimicrobial Susceptibility Testing (EUCAST; http://www.eucast.org/). ESBL production was defined as synergy between clavulanic acid and at least one of the extended-spectrum cephalosporins (ceftazidime, cefotaxime, or cefepime) or aztreonam [16]. The minimum inhibitory concentration (MIC) of the AC-cefixime combination was determined by the E-test method (AB bio Mérieux, Solna, Sweden) as described [17]. The breakpoint used for the AC-cefixime combination was the one for cefixime (1 mg/L) as specified by EUCAST.

### Statistical analysis

We compared categorical variables with chi-square or Fisher exact test and continuous variables with Mann-Whitney U tests. P<0.05 was considered statistically significant for all tests. The analysis was performed with STATA 14 (Stata Corp LP, College Station, TX).

### Ethical approval

The data collection was approved by the French National Data Protection Commission (CNIL, no. A01886-33), the Committee on the Processing of Research Information (CCTIRS, no. 13.341) and the Ethics Committee of Creteil Intercommunal Hospital. Because this is a non-interventional study, our local ethics committee and the CCTIRS approved obtaining oral consent from parents for this retrospective part of the study. Written consent was not required. The study was registered at ClinicalTrials.gov (NCT02832258).

## Results

### Baseline demographics

**Fig 1:**
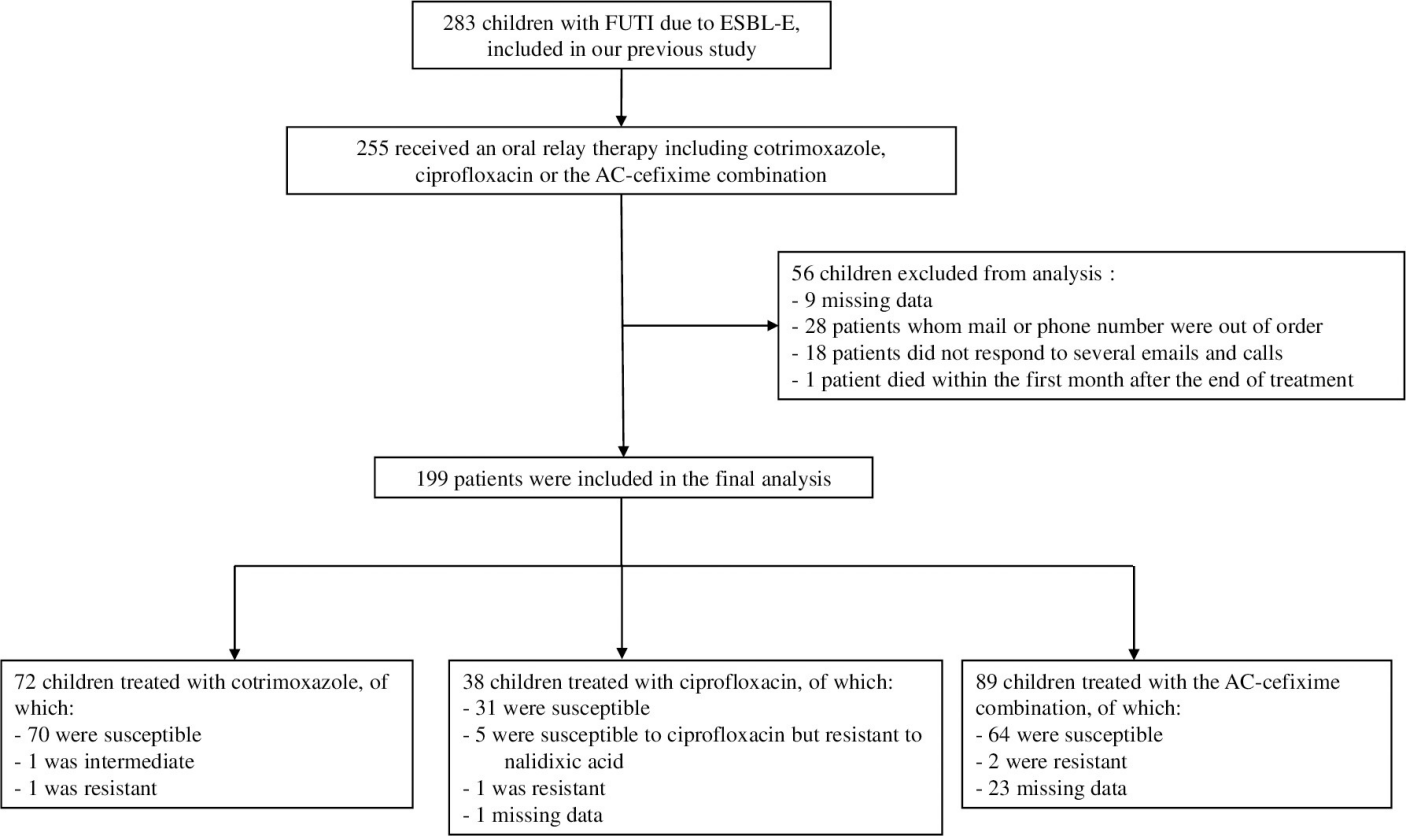
Study flow chart.

Among the 283 children included in our previously published study, we identified 255 who received cotrimoxazole, ciprofloxacin or the AC-cefixime combination as the oral relay therapy. After excluding 56 patients for the reasons mentioned above, 199 were included in the analysis: 72 (36.2%) received cotrimoxazole, 38 (19.1%) ciprofloxacin and 89 (44.7%) AC-cefixime combination (Fig 1, study flow chart).

Baseline characteristics of our study population are described in Table 1.

**Table 1:** Baseline characteristics of study population.

Characteristics	Overall population (n = 199)	Children receiving cotrimoxazole (n = 72)	Children receiving ciprofloxacin (n = 38)	Children receiving AC-cefixime combination (n = 89)
**Median age, years (range)**	1 (0.04–15.93)	0.83 (0.08–15.93)	1.38 (0.05–15.63)	1 (0.04–10.53)
**Girls, n (%)**	118 (59.3)	44 (61.1)	23 (60.5)	51 (57.3)
**Previous FUTI, n (%)**	43 (21.6)	10 (26.3)	12 (16.7)	21 (23.6)
**Urinary tract malformation, n (%)**	42 (21.1)	9 (12.5)	15 (39.5)	18 (20.2)
**Antibiotic prophylaxis, n (%)**	20 (10.1)	3 (7.9)	7 (9.7)	10 (11.2)
**Median CRP level, mg/L (range)**	79 (1–448)	80.5 (1–448)	98 (1.8–406)	69.5 (1–312)
**Bacteria involved in initial episode, n (%)**				
*Escherichia coli*	180 (90.5)	67 (93.1)	33 (86.8)	80 (89.9)
*Klebsiella*	17 (8.5)	4 (5.6)	5 (13.2)	8 (9.0)

AC: amoxicillin-clavulanic acid; FUTI: febrile urinary tract malformation; CRP: C reactive protein

Urine was collected using a collector bag (n = 87, 43.7%), midstream collection (n = 40, 20.1%) or urethral catheterization (n = 67, 33.7%). The bacteria involved in the initial FUTI were *E*. *coli* (n = 180; 90.5%), *Klebsiella pneumoniae* (n = 16, 8%), *Klebsiella oxytoca* (n = 1, 0.5%) and others (n = 2, 1%). Only 3 (1.5%) children had a positive blood culture, with the same bacteria as in urine samples.

### Antimicrobial susceptibility testing data

**Fig 2:**
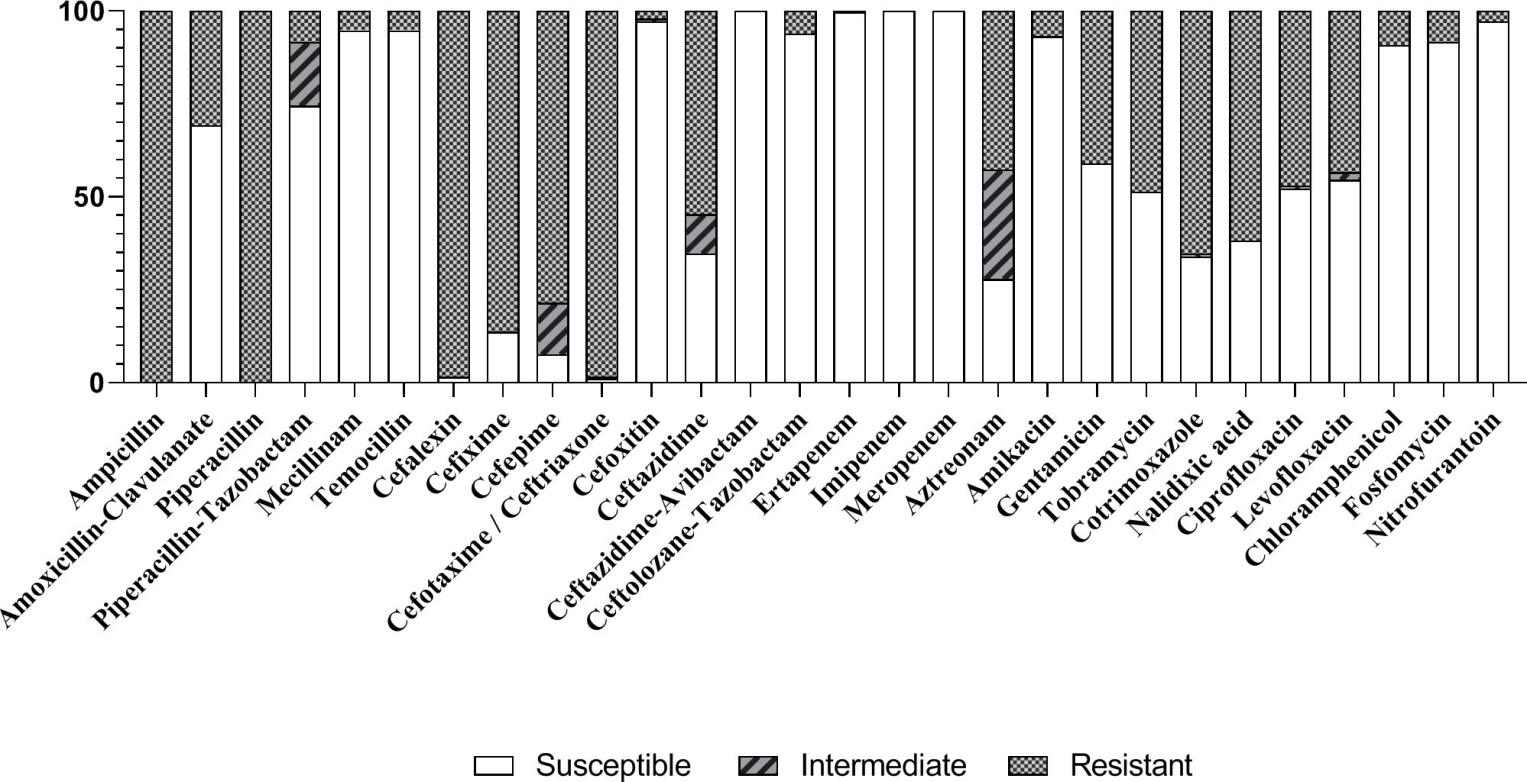
Susceptibility drug testing of the ESBL producing strains analyzed by the National Reference Center. Susceptible, resistant and intermediate results were analyzed following the latest EUCAST guidelines.

In total, 249 strains of ESBL-E identified during the first published study were sent to the NRC for further phenotypic characterization. Were included 135 of the 199 (67.8%) strains involved in this clinical study, because some centers did not send the strains to the NRC and others sent strains from 2014 up to 2019. Overall, 97.6% of the 249 strains analyzed by the NRC were susceptible to the AC-cefixime combination (MIC ≤ 1 with the E-test method). By comparison, only 33.9% of the strains were susceptible to cotrimoxazole and 52.2% to ciprofloxacin (38.2% to nalidixic acid) (Fig 2).

Among the 6 strains with a MIC > 1 mg/L to the AC-cefixime combination, all were susceptible to amikacin, and all had an effective intravenous therapeutic alternative including one of 3 drugs: cefoxitin, piperacillin-tazobactam and temocillin, which allows for carbapenems sparing. Furthermore, among the strains susceptible to the AC-cefixime combination, 96% were also susceptible to cefoxitin, 94.4% to temocillin and 73.5% to piperacillin-tazobactam.

### Initial treatment and short outcome

Overall, 177 (88.9%) children received empirical treatment with a parenteral therapy including the following antibiotics (alone or in combination): ceftriaxone/cefotaxime (n = 117, 58.8%), amikacin (n = 106, 53.3%), other aminoglycosides (n = 19, 9.5%), penems (n = 3, 1.5%) (Table 2). The others received empirical treatment with an oral antibiotic therapy including cefixime alone (n = 18; 9%), ciprofloxacin (n = 1; 0.5%), amoxicillin-clavulanate (n = 1; 0.5%) and a combination of cefixime with amoxicillin (n = 1; 0.5%) or cefixime with AC (n = 1; 0.5%). After recovery of the strain’s DST, treatment was changed to an effective intravenous antibiotic (EIA) therapy followed by the oral relay or directly to the oral relay therapy. The mean length of hospital stay (LOS) was 2.05 days (range 0–13), but about half of the patients (n = 99; 49.7%) received treatment on an outpatient basis.

**Table 2:** Treatment and clinical evolution, according to oral antibiotic relay.

Characteristics	Overall population (n = 199)	Children receiving cotrimoxazole (n = 72)	Children receiving ciprofloxacin (n = 38)	Children receiving AC-cefixime combination (n = 89)
**Empirical antibiotic therapy, n (%)**				
Parenteral 3GC	117 (58.8)	47 (65.3)	24 (63.2)	46 (51.7)
Amikacin	106 (53.3)	32 (44.4)	21 (55.3)	53 (59.6)
Oral antibiotic alone	22 (11.1)	8 (11.1)	2 (5.3)	12 (13.5)
Other aminoglycosides	19 (9.5)	5 (6.9)	4 (10.5)	10 (11.2)
Penems	3 (1.5)	-	-	3 (3.4)
**EIA, n (%)**	159 (79.9)	51 (70.8)	34 (89.5)	74 (83.1)
**Time to apyrexia, days, mean**	1.73	1.52	2.00	1.80
**LOS, days, mean**	2.05	1.73	2.42	2.15
**Duration of treatment (oral and/or intravenous) before oral relay, days, mean**	3.86	3.27	4.24	4.20
**Duration of EIA, days, mean**	2.58	1.99	2.82	2.98
**Duration of oral relay, days, mean**	8.20	8.47	8.32	7.92
**Total duration of treatment, days, mean**	12.06	11.74	12.55	12.11
**Recurrence, n (%)**	9 (4.5)	4 (5.6)	2 (5.3)	3 (3.4)

AC: amoxicillin-clavulanic acid; 3GC: third-generation cephalosporin; EIA: effective intravenous antibiotic therapy before and after receiving drug susceptibility testing; LOS: length of hospital stay

Among the 199 children included, 159 (79.9%) received an EIA therapy before oral relay: 138 (69.3%) received amikacin or another aminoglycoside, 19 (9.5%) penems and 2 (1%) piperacillin-tazobactam. The mean EIA therapy duration was 2.58 days (range 0–11) overall but differed in the 3 groups of oral relay therapy: 1.99 days (range 0–9) with cotrimoxazole, 2.82 (0–9) with ciprofloxacin and 2.98 days (0–11) with the AC-cefixime combination (Table 2). The other 40 (20.1%) patients received a non-effective oral or parenteral therapy before oral relay: 21 in the cotrimoxazole group, 15 in the AC-cefixime combination group and 4 in the ciprofloxacin group.

### Recurrence at 1 month after treatment for a FUTI due to ESBL-E

Among the 199 children included, 9 (4.5%) reported a recurrence within the first month after the end of treatment: 4 (5.6%) with cotrimoxazole, 2 (5.3%) with ciprofloxacin and 3 (3.4%) with the AC-cefixime combination (Table 2). The 3 groups did not differ in recurrence rate (p = 0.8). The recurrences were 8 FUTIs and 1 cystitis and were caused mainly by an ESBL-E: *E*. *coli* in 6 cases and *K*. *pneumonia* in 1 case. The remaining recurrences were caused by a non-ESBL producing *E*. *coli* in 1 case and an unknown pathogen in the last case (missing data). We were not able to retrieve the information on the antibiotics used to treat these recurrence because in most cases the parents did not remember it and it was not clearly recorded in the child’s “health record”.

Patients who did or did not receive an EIA therapy before oral relay did not differ in FUTI recurrence rate at 1 month after the end of treatment (7 recurrences [4.4%] vs 2 recurrences [5.0%], respectively) (Table 3).

**Table 3:** Clinical evolution comparison between children who received and those who did not receive an effective intravenous antibiotic (EIA) therapy before oral relay.

	No EIA before oral relay (n = 40)	EIA before oral relay (n = 159)
**Mean duration of antibiotic (oral and/or parenteral) before oral relay, days (range)**	2.90 (1–6)	4.11 (1–12)
**Mean time to apyrexia, days (range)**	1.33 (0–6)	1.79 (0–6)
**Mean LOS, days (range)**	0.70 (0–5)	2.38 (0–13)
**Recurrence, n (%)**	2 (5.0)	7 (4.4)

EIA: effective intravenous antibiotic therapy; LOS: length of hospital stay

Notably, 4 of the 9 (44.4%) patients with a recurrence had an underlying urinary tract malformation as compared with 42 (21.1%) in the overall population: 18 (20.2%) of the AC-cefixime group, 9 (12.5%) of the cotrimoxazole group and 15 (39.5%) of the ciprofloxacin group. In addition, 4 of the 9 recurrences (44.4%) were related to an initial FUTI caused by *Klebsiella* (*pneumoniae* and o*xytoca*) strains, the rest to *E*. *coli*.

## Discussion

To our knowledge, this is the first study comparing oral antibiotic relay in FUTI due to ESBL-E in children in terms of recurrence at 1 month after the end of treatment. We found no differences in the recurrence rate whatever the oral antibiotic relay used (cotrimoxazole, ciprofloxacin or the AC-cefixime combination). In addition, 97.6% of the ESBL-E strains analyzed by the NRC were susceptible to the AC-cefixime combination. This combination could be an interesting choice for oral relay, all the more so as few therapeutic options are available and only half of the same strains were susceptible to ciprofloxacin (and nalidixic acid) and a third to cotrimoxazole.

Our study used data from a large national prospective study that described FUTI due to ESBL-E in children over a 3-year period. It represents 199 of the 283 (70%) patients analyzed and previously published [14]. The short-term evolution of these infections was similar when they were treated with effective or ineffective empirical therapy [14], but we showed a similar outcome in recurrence at 1 month after the end of treatment whatever the oral antibiotic relay used.

In our cohort of children, 159 (79.9%) received an EIA therapy as first-line treatment before oral relay (adapted to DST); of these, 19 (9.5%) received carbapenems. Patients who received an EIA therapy before oral relay and those who did not, had a similar recurrence rate at 1 month after the end of treatment. Hence, the positive outcome of FUTI due to ESBL-E that we report here, regardless of the antibiotic therapy received, supports a spare of carbapenems in treating these infections.

We also found that the mean duration of the EIA therapy before oral relay significantly differed among the 3 treatment groups. This finding may have affected the outcome, given that the AC-cefixime combination group received an EIA therapy for longer than the 2 other groups. However, in our opinion, this result only reflects the time needed to test the strain’s susceptibility to the AC-cefixime combination (with the E-test method) after receiving the initial antibiogram results showing resistance to cotrimoxazole and ciprofloxacin.

Furthermore, the choice of the oral relay antibiotic does not seem to be the main determinant for recurrence because significantly more patients had an underlying urinary tract malformation in the recurrence group than in the overall population (44.4% vs 21.1%). These malformations are known to be major contributors for recurrent FUTI. Likewise, we noted a significant higher percentage of ESBL-producing *Klebsiella* (*pneumoniae* and *oxytoca*) strains causing the initial FUTI in patients with recurrence than in the overall population (44.4% vs 8.5%). *Klebsiella sp*. strains are usually less uropathogenic than *E*. *coli* strains but are more frequently involved in FUTI in children with urinary tract malformations.

The 2 major strengths of our study are the large number of patients with FUTI due to ESBL-E included from multiple centers throughout France and the analysis by the NRC of a large representative sample of the strains involved in this study (135/199; 67.8%).

Other studies have investigated the clinical effectiveness of an oral third-generation cephalosporin combined with AC and found high clinical cure rates but in a smaller number of adults [18, 19]. This situation emphasizes the need for larger prospective studies in children.

The main limitation of our study is that the diagnosis of pyelonephritis was not certain. Indeed, urine was collected by bladder probing or a collector bag as allowed by French recommendations. To have a definite diagnosis, a renal scintigraphy is recommended but limits the feasibility of such studies.

Another limitation might be the use of the E-test method for the DST of the ESBL-producing strains to the AC-cefixime combination because it is not standardized. However, several other studies, using various non-standardized methods [19–21], including the E-test [22], have shown *in vitro* synergy between third-generation cephalosporins and clavulanate. We chose the E-test because it can detect *in vitro* synergy between cefixime and clavulanate and for its ease of use in clinical laboratory settings [22]. In fact, our study reports a large number of strains (n = 249) tested by the NRC with the E-test method for susceptibility to the AC-cefixime combination, which was also used by many centers throughout France before treatment with this combination.

Although this was a retrospective study whose results need to be confirmed by prospective studies, our results support the use of the AC-cefixime combination for oral relay therapy of FUTI due to ESBL-E in children, especially if the strain is resistant to cotrimoxazole or fluoroquinolones. This solution could help many physicians in an era of increasing resistance to usual antibiotics, allowing for shorter parenteral treatment time and shorter hospitalizations.

## Conclusions

The AC-cefixime combination represents an interesting therapeutic option for oral relay treatment of FUTI due to ESBL-E as the recurrence rate at 1 month after the end of treatment was the same when compared to cotrimoxazole and ciprofloxacin, and most ESBL-E strains remained susceptible to this unorthodox combination.

## References

[1] Dayan N, Dabbah H, Weissman I, Aga I, Even L, Glikman D. Urinary Tract Infections Caused by Community-Acquired Extended-Spectrum β-Lactamase-Producing and Nonproducing Bacteria: A Comparative Study. J Pediatr. 2013; 163: 1417–1421. doi: 10.1016/j.jpeds.2013.06.078 23919903

[2] Subcommittee on Urinary Tract Infection, Steering Committee on Quality Improvement and Management, Roberts KB. Urinary tract infection: clinical practice guideline for the diagnosis and management of the initial UTI in febrile infants and children 2 to 24 months. Pediatrics. 2011; 128: 595–610. doi: 10.1542/peds.2011-1330 21873693

[3] Shaikh N, Morone NE, Bost JE, Farrell MH. Prevalence of urinary tract infection in childhood: a meta-analysis. Pediatr Infect Dis J. 2008; 27: 302–308. doi: 10.1097/INF.0b013e31815e4122 18316994

[4] Hanna-Wakim RH, Ghanem ST, El Helou MW, Khafaja SA, Shaker RA, Hassan SA, et al. Epidemiology and characteristics of urinary tract infections in children and adolescents. Front Cell Infect Microbiol. 2015; 5: 45. doi: 10.3389/fcimb.2015.00045 26075187 PMC4443253

[5] Zorc JJ, Kiddoo DA, Shaw KN. Diagnosis and Management of Pediatric Urinary Tract Infections. Clin Microbiol Rev. 200; 18: 417–422. doi: 10.1128/CMR.18.2.417-422.2005 15831830 PMC1082801

[6] Pitout JD, Laupland KB. Extended-spectrum β-lactamase-producing Enterobacteriaceae: an emerging public-health concern. Lancet Infect Dis. 2008; 8: 159–166. doi: 10.1016/S1473-3099(08)70041-0 18291338

[7] Livni G, Ashkenazi S. Treatment of resistant bacterial infections in children: thinking inside and outside the box. Adv Exp Med Biol. 2013; 764: 123–132. doi: 10.1007/978-1-4614-4726-9_9 23654061

[8] Fan N-C, Chen H-H, Chen C-L, Ou LS, Lin TY, Tsai MH, et al. Rise of community-onset urinary tract infection caused by extended-spectrum β-lactamase-producing Escherichia coli in children. J Microbiol Immunol Infect. 2014; 47: 399–405. doi: 10.1016/j.jmii.2013.05.006 23834784

[9] National Institute for Health and Clinical Excellence (NICE). Urinary tract infection in children. Available at: https://www.nice.org.uk/Guidance/cg54 [Last Accessed 2 April 2017].

[10] Roberts KB, Downs SM, Finnell SM, et al. American Academy of Pediatrics Subcommittee on Urinary Tract Infection, Steering Committee on Quality Improvement and Management. Urinary tract infection: clinical practice guideline for diagnosis and management of the initial UTI in febrile infants and children 2 to 24 months. Pediatrics. 2011; 128: 595e610. doi: 10.1542/peds.2011-1330 21873693

[11] Stein R, Dogan HS, Hoebeke P, Kočvara R, Nijman RJ, Radmayr C, et al. European Association of Urology and European Society for Pediatric Urology. Urinary tract infections in children: EAU/ESPU guidelines. Eur Urol. 2015; 67: 546e58. doi: 10.1016/j.eururo.2014.11.007 25477258

[12] Strohmeier Y, Hodson EM, Willis NS, Webster AC, Craig JC. Antibiotics for acute pyelonephritis in children. Cochrane Database Syst Rev. 2014; 28, CD003772. doi: 10.1002/14651858.CD003772.pub4 25066627 PMC10580126

[13] Poey N, Madhi F, Biscardi S, Béchet S, Cohen R. Aminoglycosides Monotherapy as First-Line Treatment for Febrile Urinary Tract Infection in Children. Pediatr Infect Dis J. 2017; 36: 1104–1107. doi: 10.1097/INF.0000000000001636 28498305

[14] Madhi F, Jung C, Timsit S, Levy C, Biscardi S, Lorrot M, et al. Febrile urinary-tract infection due to extended-spectrum beta-lactamase-producing Enterobacteriaceae in children: A French prospective multicenter study. PloS One. 2018; 13: e0190910. doi: 10.1371/journal.pone.0190910 29370234 PMC5784917

[15] Cohen R, Raymond J, Faye A, Gillet Y, Grimprel E, Société française de pédiatrie; Société de pathologie infectieuse de langue française. [Management of urinary tract infections in children. Recommendations of the Pediatric Infectious Diseases Group of the French Pediatrics Society and the French-Language Infectious Diseases Society]. Arch Pediatr. 2015; 22: 665–671. doi: 10.1016/j.arcped.2015.03.016 25934607

[16] M100Ed30 | Performance Standards for Antimicrobial Susceptibility Testing, 30th Edition [Internet]. Clinical & Laboratory Standards Institute. [cited 2021 Jan 25]. Available from: https://clsi.org/standards/products/microbiology/documents/m100/

[17] Drieux L, Brossier F, Sougakoff W, Jarlier V. Phenotypic detection of extended-spectrum beta-lactamase production in Enterobacteriaceae: review and bench guide. Clin Microbiol Infect. 2008; 14: 90–103. doi: 10.1111/j.1469-0691.2007.01846.x 18154532

[18] Cohen Stuart J, Leverstein-Van Hall M, Verlind J, Mulder F, Scharringa J, et al. Ceftibuten plus amoxicillin-clavulanic acid for oral treatment of urinary tract infections with ESBL producing E. coli and K. pneumoniae: a retrospective observational case-series. Eur J Clin Microbiol Infect Dis. 2018; 37: 2021–2025. doi: 10.1007/s10096-018-3338-z 30117050

[19] Al-Tamimi M, Abu-Raideh J, Albalawi H, Shalabi M, Saleh S. Effective Oral Combination Treatment for Extended-Spectrum Beta-Lactamase-Producing Escherichia coli. Microb Drug Resist. 2019; 25: 1132–1141. doi: 10.1089/mdr.2019.0065 31107146

[20] Livermore DM, Hope R, Mushtaq S, Warner M. Orthodox and unorthodox clavulanate combinations against extended-spectrum beta-lactamase producers. Clin Microbiol Infect. 2008; 14: 189–193. doi: 10.1111/j.1469-0691.2007.01858.x 18154546

[21] Campbell JD, Lewis JS, McElmeel ML, Fulcher LC, Jorgensen JH. Detection of favorable oral cephalosporin-clavulanate interactions by in vitro disk approximation susceptibility testing of extended-spectrum-Beta-lactamase-producing members of the enterobacteriaceae. J Clin Microbiol. 2012; 50: 1023–1026. doi: 10.1128/JCM.06248-11 22170910 PMC3295132

[22] Bingen E, Bidet P, Birgy A, Sobral E, Mariani P, Cohen R. In vitro interaction between cefixime and amoxicillin-clavulanate against extended-spectrum-beta-lactamase-producing Escherichia coli causing urinary tract infection. J Clin Microbiol. 2012; 50: 2540–2541. doi: 10.1128/JCM.00526-12 22535978 PMC3405603

